# Remotely supervised ultrasound‐guided peripheral intravenous cannulation training: A prospective cohort study examining success rates and patient experience

**DOI:** 10.1002/ajum.12318

**Published:** 2022-09-16

**Authors:** Nathan Peters, Joel Thomas, Christine Woods, Claire Rickard, Nicole Marsh

**Affiliations:** ^1^ Department of Anaesthesia and Perioperative Medicine Royal Brisbane and Women's Hospital Brisbane Queensland Australia; ^2^ University of Queensland Brisbane Queensland Australia; ^3^ Department of Surgery University of Melbourne Melbourne Victoria Australia; ^4^ School of Nursing and Midwifery Griffith University Brisbane Queensland Australia; ^5^ Alliance for Vascular Access Teaching and Research Group Griffith University Brisbane Queensland Australia; ^6^ Metro North Hospitals and Health Service Brisbane Queensland Australia

**Keywords:** catheterisation, difficult vascular access, education, peripheral, ultrasound guidance

## Abstract

**Introduction:**

Ultrasound‐guided peripheral intravenous cannulation (USGPIVC) benefits patients with difficult intravenous access (DIVA) through visualising otherwise non‐visible and non‐palpable veins. Supervised live‐case training is an important component of learning this skill, but supervisor availability can present a barrier limiting or delaying staff completing their training.

**Aims:**

The aim of this study was to determine the first‐attempt success rate of newly trained USGPIVC inserters using remote supervision and timely written feedback based on app‐based screen recordings taken during insertion. Secondary aims were overall procedural success, and inserter and patient experiences.

**Methods:**

This study is an observational cohort study carried out between October and December 2021. Fourteen newly trained junior medical officers (JMOs) were eligible to utilise USGPIVC on a minimum of five consenting patients while simultaneously recording the ultrasound screen during insertion to capture their technique. Feedback was generated following expert review of these recordings against a standardised feedback tool.

**Results:**

Average first‐attempt success was 71% (n = 72) in the 102 patients recruited. The average time for JMOs to receive feedback was 30 h, and 13 JMOs (93%) felt well supported and completed the remote training pathway. The majority of patients were female (n = 59; 58%), were aged 41–80 years (n = 75; 74%) and had ≥2 risk factors for DIVA (n = 57; 56%).

**Conclusions:**

First‐attempt success rates were similar when comparing remote supervision used in this study to direct supervision used by other studies.This finding supports incorporating remote supervision into training guidelines for USGPIVC as an alternative method of supervision, particularly when supervisor availability is limited.

## Introduction

### Background

Peripheral intravenous catheters (PIVCs) are the most common invasive medical device in hospitalised patients, and in Australia and New Zealand, the majority are inserted by medical staff.^1^, ^2^, ^3^ However, difficult peripheral intravenous cannulation has been reported to occur in 12–39% of hospitalised adult patients, rising to 59% in those with complex medical needs.^4^, ^5^, ^6^, ^7^ For patients, this results in multiple attempts at insertion leading to discomfort, delays in medical therapy and increased complications (*i.e*. infections and phlebitis).^8^, ^9^, ^10^

Ultrasound‐guided peripheral intravenous cannulation (USGPIVC) benefits difficult intravenous access (DIVA) patients by allowing visualisation of otherwise non‐visible and non‐palpable veins, thereby increasing first‐attempt cannulation success.^11^, ^12^ Face‐to‐face short courses of between 2‐ and 3‐hour duration are an effective and favoured way for novice ultrasound users to learn initial USGPIVC skills.^13^ However, without further live‐case supervision less than half of newly trained staff go onto perform USGPIVC, indicating supervised clinical practice is an important component for skill development.^14^ It is therefore recommended that a period of clinical supervision be incorporated into USGPIVC training pathways.^14^, ^15^ Clinical supervision improves ongoing clinical practice and patient care by fostering a culture that is educational, self‐critical and patient‐focussed.^16^ However, one of the greatest recognised barriers in delivering supervision, not only in ultrasound training pathways, is the lack of availability of experienced clinicians to be at the patient's bedside in order to provide this supervision.^17^, ^18^ So, while ongoing supervised practice is designed to improve skill translation and competency, ensuring timely access to expert supervision can instead present itself as a potential barrier.

Ultrasound‐guided peripheral intravenous cannulation insertion requires the inserter to focus their attention on the ultrasound screen, follow the needle tip on the ultrasound image and guide the PIVC into the vein in real time. Ault *et al*.^19^ defined USGPIVC competency as: being able to select the right depth, gain and target vessel; properly positioning the target vein in the centre of the ultrasound screen; correctly aligning the needle tip in the middle of the probe over the target vein; tracking the catheter through the skin, soft tissue and vein wall; and finally cannulating the vein successfully and achieving a bullseye image. All these components can be observed on ultrasound images which, if recorded at the time of insertion, can be reviewed and appraised retrospectively by an expert using a standardised feedback form.

The ability to remotely supervise and provide timely standardised feedback to newly trained USGPIVC practitioners could enable staff to overcome the practical barrier of direct supervision during live‐case training. This research investigated whether remote supervision could be an alternative to the current labour‐intensive and costly requirement of having a physically present supervisor during the live‐case training component of USGPIVC training.

### Study aim

The aim of this study was to determine the first‐attempt cannulation success rate of newly trained USGPIVC inserters with ultrasound using remote supervision and timely written feedback.

## Methods

### Study design and setting

A prospective observational cohort study was undertaken between October and December 2021 at the Royal Brisbane and Women's Hospital in Queensland, Australia. This study was approved by the participating hospital's Human Research Ethics Committee (Ref No.: LNR/2021/QRBW/76402) and follows the STROBE guidelines for reporting of observational studies in health care.^20^

### Participants

#### Junior medical officers

Junior medical officers (JMOs) competent in landmark/palpation‐based PIVC insertion were eligible to undertake a standardised training pathway (Table 1) for USGPIVC if they met the following criteria: postgraduate year (PGY) ≥ 2; rostered to a minimum of five continuous full‐time weeks working after hours (Mon–Fri 21:30–08:00; Sat–Sun 20:30–08:00) attending to the medical needs of inpatients in medical and surgical wards; had no formal ultrasound qualification; and had performed <5 previous USGPIVC insertions. An expression of interest email was sent to all 21 JMOs rostered to after‐hours work for the period of October to December 2021. Written informed consent to participate in the study was obtained following the JMOs response to the expression of interest email. Enrolled JMOs were informed at the commencement of the requirement to complete a minimum of five remote supervised insertions during their five‐week after‐hours term and be successful in at least three insertions to be deemed able to practice independently. JMOs were instructed during training that they were only able to cannulate veins of the upper extremity.

**Table 1:** Ultrasound PIVC insertion training pathway undertaken by JMOs

USGPIVC training pathway
Online theoretical learning – 1 hour Successful completion of a self‐directed USGPIVC online theory aimed at teaching novice ultrasound users the required skills. (Clinical Skills Development Service, Queensland, USGPIVC online course)
2 Face‐to‐face simulated practical training session – 2 hours Orientation to the study aims, protocol, patient recruitment and consent. Introduction to using the Philips Lumify® (Netherlands) linear ultrasound device and the Galaxy Samsung® tablet (South Korea) as well as storage and access. Simulation‐based practical training on vascular access simulators (Blue Phantom® (Canada)). Assessment station – each participant was required to perform one mock patient encounter and demonstrate USGPIVC insertion on a simulated arm (Blue Phantom®) with sign off by a study investigator.
3 Remotely supervised practice Requirement of a minimum of five insertions and successful insertion in at least three patients to be able to practice independently.

JMOs, junior medical officers; PIVC, peripheral intravenous catheters; USGPIVC, ultrasound‐guided peripheral intravenous cannulation.

#### Patients

Inpatients ≥18 years of age were eligible if they required a PIVC for medical reasons and were able to provide written informed consent for study participation. JMOs recruited patients at their discretion during their after‐hours work and could be low‐, moderate‐ or high‐risk DIVA patients.

### Variables

#### Primary outcome

An attempt was defined as a single percutaneous needle puncture, regardless of the amount of subcutaneous exploration, and success was defined as being able to flush the newly inserted PIVC with 10 ml of 0.9% saline without signs of infiltration.^21^ If successful cannulation was achieved on the first attempt, this was considered as first‐attempt success.

#### Secondary outcomes

The number of ultrasound‐guided attempts per patient encounter was also recorded. After an unsuccessful first attempt, a new attempt could be undertaken by the JMO by firstly localising a vein, followed by a new percutaneous puncture. A procedure was defined as all attempts using ultrasound by the JMO at establishing intravenous access in a given patient with the number of attempts limited to a maximum of two based on hospital policy. The number of newly trained JMOs completing five USGPIVCs was also assessed from insertion data. JMO's self‐reported confidence and satisfaction with the training pathway were assessed at the end of the study period. Patient experience and PIVC outcomes were also measured including comfort, patient perception of their level of difficulty and inserter skill and PIVC patency.

### Data sources

Data were collected and collated using the REDCap software (Research Electronic Data CAPture, Vanderbilt).^22^ For each patient encounter, irrespective of successful placement, the JMO recorded their name, patient A‐DIVA risk factors and PIVC insertion details into the research database. A‐DIVA rating scale included a known history of DIVA, expectation of failed first attempt or DIVA, inability to identify a dilated vein by visualising or palpating the upper extremity and largest dilated vein being <3 mm in diameter.^8^

Immediately before commencing the USGPIVC attempt, JMOs started the inbuilt screen recording application (DU Recorder®) on the Samsung Galaxy® tablet running the Philips Lumify® linear ultrasound transducer. Screen recordings were downloaded off the tablet and stored in a secure location for the CIs (NP and JT, both USGPIVC experts with postgraduate ultrasound qualifications and >500 insertions) to view and provide feedback in the research database.

Prior to study commencement, the research team developed a standardised feedback tool by reviewing previously published literature and identifying important components for training and procedural success.^15^, ^19^, ^23^, ^24^ This highlighted three key commonly identified areas of practice needing to be demonstrated to achieve competency. These three main areas were ultrasound machine set‐up, appropriate vein selection and needle tip tracking, with subheadings within each category aimed at maximising cannulation success. (Appendix A or https://metronorth.health.qld.gov.au/uploads/usgpivcfeedback.pdf) Ultrasound screen recordings were matched to patient encounter data recorded by JMOs at the time of insertion, and feedback was returned to JMOs *via* email within 48 h. Following the review of the feedback, JMOs were required to complete a feedback acknowledgement form indicating their satisfaction with the feedback (1 = not satisfied to 5 = very satisfied), and whether they would like to be contacted by a supervisor.

A member of the research team attempted to review enrolled patients the following day or, when not appropriate, as soon as possible. Willing patients completed the Patient Reported Experience Measure (PREM) questionnaire based on the research by Cuthbertson and Ashton.^25^ This is related to the last PIVC insertion and included the number of attempts, difficulty of insertion, inserter skill, complications and importance of research into PIVC management.

At the conclusion of the study timeframe, JMOs were asked to give feedback on their confidence relating to their USGPIVC skill and overall satisfaction (1 = strongly disagree to 5 = strongly agree) with the remote supervision aspect of the training pathway.

### Study size

Prior to study commencement, sample size was pre‐determined to be a minimum of 50 USGPIVC insertion attempts based on sample size recommendations for feasibility trials.^26^ This equated to ten junior doctors each with a minimum of five patient care episodes.

### Statistical analysis

Observational endpoints were analysed by the investigator team with quantitative data identified as continuous variables. No inferential statistical analysis was planned.

### Ethics approval

This study was approved by the participating hospital's Human Research Ethics Committee (Ref No: LNR/2021/QRBW/76402). Informed written consent was obtained from JMOs and patients recruited in the study.

## Results

### Junior medical officers characteristics

Of the 21 JMOs emailed study information, 15 expressed interest and 14 met the eligibility criteria. One JMO was excluded as they had already performed >50 USGPIVC insertions. Most participants were male (n = 8; 57%), with an average age of 30 and were two (n = 11; 79%) or three (n = 3; 21%) years postgraduate.

### Patient characteristics

Of the 102 patients recruited, 59 (58%) were female and most were from medical wards (n = 60; 59%). Ages ranged from 18 to 92 years with the 75 (74%) patients aged 41–80 years. Patients were categorised according to the number of risk factors as per the A‐DIVA rating scale, with 45 (44%) at low DIVA risk (0–1 risk factors), 39 (38%) at moderate DIVA risk (2–3 risk factors) and 18 (18%) at high DIVA risk (4–5 risk factors).

### Descriptive data

Of the 14 enrolled JMOs, 13 performed five or more USGPIVC insertions during their 5 weeks of after‐hours work. After completing face‐to‐face training, one recruited JMO did not undertake any USGPIVC insertion attempts during the study due to a large proportion of patients encountered having established central venous access.

### Outcome data

#### Primary outcome

Average first insertion success by the newly trained JMO using ultrasound was 71% (n = 72) in the 102 patients encountered. Of the 27 second attempts, 13 (48%) were successful giving an overall procedural success rate of 83% (n = 85) (Table 2).

**Table 2:** First‐attempt and procedural success rates for each participant

Participant	Number of patients (procedures)	Total number of attempts	First‐attempt^a^ success N (%)	Procedural^b^ success N (%)
A	9	15	1 (11%)	2 (22%)
B	6	6	6 (100%)	6 (100%)
C	9	11	7 (78%)	9 (100%)
D	12	14	9 (75%)	10 (83%)
E	6	6	6 (100%)	6 (100%)
F	5	6	4 (80%)	5 (100%)
G	6	9	3 (50%)	3 (50%)
H	16	24	8 (50%)	14 (88%)
I	8	8	8 (100%)	8 (100%)
J	5	6	4 (80%)	4 (80%)
K	5	8	2 (40%)	3 (60%)
L	6	6	6 (100%)	6 (100%)
M	9	10	8 (89%)	9 (100%)

^a^ Attempt: a single percutaneous needle puncture, regardless of the amount of subcutaneous exploration.

^b^ Procedure: all attempts needed to achieve successful intravenous access in each patient.

Feedback from ultrasound screen recordings was provided on 100 of 102 patient encounters. Two screen recordings were missed because the JMO did not commence the screen recording application correctly. Results from the feedback are shown in Table 3. In all 100 cases, JMOs reviewed their feedback forms and in 94% of cases felt completely satisfied with the feedback given.

**Table 3:** Overall frequency each variable was achieved from all feedback forms

	Percentage achieved for all insertions (N = 100)
**Ultrasound machine set‐up**	
US preset selected (vascular/superficial)	98%
Depth of US field ≤ 3 cm	93%
Gain appropriate – enough to see surrounding tissue plains as white and centre of vessel as black	94%
**Vein selection**	
Vessel easily identified as a vein and not an artery	100%
Vessel size (ideally >4 mm diameter)	37%
Vessel selected not too superficial – (>5 mm to anterior vessel wall)	59%
Vessel selected not too deep – (<20 mm to anterior vessel wall)	100%
Some part of vein touching centre line (or mid‐screen if no centre line used) when the needle first visualised	96%
**Needle tip tracking**	
Easily seen outside the vessel during approach to vessel	83%
Gradually redirected and moved inside the vessel	75%
Hyperechoic needle tip centre in the vein at end of recording – Bullseye sign seen	66%

A total of 78 (76%) patient questionnaires were completed. Reasons for not completing the questionnaire included being absent from the ward (i.e. discharged) or feeling too unwell to give feedback. Patients reported their mean PIVC insertion difficulty as 7.5 out of 10 based on previous cannulation experiences. Patient perception of JMO USGPIVC insertion skill was high, with a mean score of 8.4 out of 10. Complications arising from the USGPIVC inserted by the JMO were noted by 28 (35%) of patients questioned. PIVC failure (n = 13; 16%) was most commonly followed by pain on insertion (n = 7; 9%), fluid leak (n = 4; 5%), bruising (n = 3; 4%) and uncomfortable location (n = 1; 1%). Patient perception of the importance of continuing research into PIVC insertion and management was high, with a mean score of 9.0 of 10.

Average time from USGPIVC insertion to feedback completion was 30 hours with one JMO contacting the supervisors for additional assistance during the study period. All 14 JMOs completed a satisfaction survey at the end of the training period, and their responses are shown in Table 4.

**Table 4:** JMO feedback upon completion of the training pathway

JMO feedback on training pathway	Agree/Strongly Agree (%) (N = 14)
I felt the training course and the orientation session gave me enough confidence to commence live training without the presence of a supervisor at the bedside	13 (93%)
There were enough opportunities to perform USGPIVC on patients during after‐hours work	12 (86%)
The ultrasound machine provided for the study was easy to access after hours	13 (93%)
I felt my learning was well supported by the remote supervision provided in this study	13 (93%)
I was able to use the feedback from the supervisors' reports to help develop my skill in USGPIVC further	13 (93%)
I found that having a needling phantom available for extra simulation‐based practice was useful	4 (29%)
I would prefer to learn through this method of remote supervision than having a supervisor immediately present at the bedside	7 (50%)
I would recommend this style of remotely supervised practice to a colleague who wanted to learn the skill of USGPIVC	13 (93%)
After completing this study, I feel confident in using ultrasound in patients with Difficult IV Access from now on	12 (86%)

JMO, junior medical officers; USGPIVC, ultrasound‐guided peripheral intravenous cannulation.

## Discussion

Our results are important because they demonstrate the success of an USGPIVC training pathway incorporating remotely supervised practice. Most JMOs (n = 12; 92%) met the requirements to progress to unsupervised practice with only one being referred for additional training and direct supervision. Supervised practice fosters further learning and allows newly trained staff to feel supported while demonstrating safe and sufficient quality practice before transitioning to unsupervised practice.^16^ For this study, we required at least three successful insertions over a minimum of five remotely supervised attempts to be deemed able to practice independently. This was based on other studies incorporating three to five supervised insertions as part of training.^27^, ^28^, ^29^, ^30^ Previous research has demonstrated a steep learning curve when bedside supervision is employed, with Stolz *et al* predicting a first‐attempt success rate of >70% following four attempts and van Loon demonstrating a first‐attempt success rate of 81% over the first 10 directly supervised insertions.^21^, ^31^ Our first‐attempt success rate of 71% after an average of eight insertions is comparable with these other published first‐attempt success rates utilising direct bedside supervision. This indicates that remote supervision results in similar first‐attempt success rates when compared to direct supervision for newly trained staff undertaking USGPIVC.

Collation of screen recording feedback gave insight into the common difficulties encountered by novice USGPIVC inserters. Owing to default machine settings, this element was overall very well achieved by JMOs. A commonly encountered problem however was the selection of superficial and small diameter veins requiring precise needle tip movements, often difficult for the novice. Needle tip tracking, although impacted by vein selection, demonstrated a gradual decline in achievement through identifying the tip outside the vessel (83%), manoeuvring it inside the vessel (75%) and being able to centre the needle tip within the vessel at the conclusion of the procedure (66%). Needle tip tracking is a key component of USGPIVC, and novices need to focus on developing this skill during their early learning.^32^ Future training programmes should therefore place more emphasis on vein size and depth as well as needle tip tracking as this may improve first insertion success rates.

Importantly, 83% of patients in this majority DIVA cohort had a PIVC inserted with the aid of ultrasound. As per the A‐DIVA risk assessment tool described by van Loon *et al*, without ultrasound patients at moderate DIVA risk were reported to have a 63% first‐attempt success rate and high DIVA risk a 6% first‐attempt success rate.^8^ In this study, the first‐attempt success rate when using ultrasound guidance was 80% (31 of 39) in moderate DIVA risk patients and 78% (14 of 18) in high DIVA risk patients. This highlights the benefits that introducing ultrasound training with remote supervision has for DIVA patients, supervisors and institutions. When staff do not have access to ultrasound after hours, DIVA patients incur delays in medical therapy through multiple uncomfortable failed cannulation attempts.^10^ However, in this study patients with DIVA potentially avoided the associated treatment delays and discomfort from failed attempts at PIVC insertion. Supervisors benefit from this system of remote supervision by eliminating the need to attend to the patient bedside to provide direct supervision allowing them to continue with other clinical duties while also expanding their oversight into the after‐hours timeframe, a time of day when direct supervision is often impossible. Institutional benefit was likely also generated through the more efficient use of staff time and organisational resources.

### Limitations and generalisability

A limitation of this study is the small sample size, and it was undertaken in a major metropolitan teaching hospital. More motivated JMOs may have been recruited through the expression of interest process, and patients recruited by them may have been subject to some selection bias. Reviewing screen recordings did not allow for observation of some aspects of insertion such as aseptic technique. However, aseptic technique was incorporated into simulation‐based training and required to be demonstrated before the beginning of live‐case training. Simulation‐based training in aseptic technique for PIVC insertion has been previously demonstrated to improve technique and knowledge retention.^33^

Supervisors and JMOs were contained within the same hospital, and results may not be generalisable to environments where both parties are not collocated. Also uncertain is the most beneficial timeframe to provide feedback to newly trained staff. During this study, instructors of the short‐course training programme were also those providing ongoing review and supervision, and results might differ if this was not the case. This study was conducted in a single centre operating within local policies (*i.e*. JMOs inserting PIVCs after hours), so results may not be transferrable to other centres or other cohorts of healthcare staff. However, a strength of this study was that decision‐making around PIVC need, maintenance and removal were, according to the treating medical team, potentially making results more generalisable to other healthcare settings.

## Conclusion

This study demonstrates that remote supervision during ultrasound‐guided peripheral intravenous cannulation (USGPIVC) training results in similar first‐attempt success rates when compared to direct supervision used in other studies, and that this training model is well received by staff and patients. Expanding the ability to supervise newly trained staff who manage difficult intravenous access patients where the availability of supervision is limited results in improvements in staff training and better patient care. As a result, our study supports incorporating remote supervision into training guidelines for USGPIVC as an alternative to the traditional methods of supervision which rely on supervisors to be physically present at the bedside.

## Author contributions

NP involved in conceptualisation, methodology, project administration, investigation, supervision, writing – original draft and reviewing and editing. JT and CW involved in conceptualisation, methodology, project administration, investigation and writing – review and editing. CR and NM involved in supervision and writing – review and editing.

## Authorship declaration

The authors acknowledge that their listing conforms to the AJUM authorship policy. They have all reviewed the manuscript and are in agreement with the contents of the manuscript.

## Funding

No funding was received for the conduct of this study.

## Conflicts of interest

NP and JT are course facilitators of the Ultrasound Guided Peripheral Intravenous Cannulation course at the Clinical Skills Development Service (MetroNorth Hospital and Health Service). CW has no conflicts of interest to declare. CR's employer (Griffith University or The University of Queensland) has received investigator‐initiated research grants or consultancy payments on her behalf. On behalf of NM, Griffith University and The University of Queensland have received investigator‐initiated research grants or consultancy payments.

## Data Availability

Individual participant data will not be available from this study.
